# Impact of Xpert MTB/RIF Testing on Tuberculosis Management and Outcomes in Hospitalized Patients in Uganda

**DOI:** 10.1371/journal.pone.0048599

**Published:** 2012-11-06

**Authors:** Christina Yoon, Adithya Cattamanchi, J. Lucian Davis, William Worodria, Saskia den Boon, Nelson Kalema, Winceslaus Katagira, Sylvia Kaswabuli, Cecily Miller, Alfred Andama, Heidi Albert, Pamela Nabeta, Christen Gray, Irene Ayakaka, Laurence Huang

**Affiliations:** 1 Division of Pulmonary and Critical Care Medicine, San Francisco General Hospital, University of California San Francisco, San Francisco, California, United States of America; 2 Curry International Tuberculosis Center, San Francisco General Hospital, University of California San Francisco, San Francisco, California, United States of America; 3 HIV/AIDS Division, San Francisco General Hospital, University of California San Francisco, San Francisco, California, United States of America; 4 Department of Medicine, Faculty of Medicine, Makerere University, Kampala, Uganda; 5 Uganda Ministry of Health, Kampala, Uganda; 6 Makerere University-University of California San Francisco Research Collaboration, Kampala, Uganda; 7 Foundation for Innovative New Diagnostics, Kampala, Uganda; 8 Foundation for Innovative New Diagnostics, Geneva, Switzerland; University of Cape Town, South Africa

## Abstract

**Rationale:**

The clinical impact of Xpert MTB/RIF for tuberculosis (TB) diagnosis in high HIV-prevalence settings is unknown.

**Objective:**

To determine the diagnostic accuracy and impact of Xpert MTB/RIF among high-risk TB suspects.

**Methods:**

We prospectively enrolled consecutive, hospitalized, Ugandan TB suspects in two phases: baseline phase in which Xpert MTB/RIF results were not reported to clinicians and an implementation phase in which results were reported. We determined the diagnostic accuracy of Xpert MTB/RIF in reference to culture (solid and liquid) and compared patient outcomes by study phase.

**Results:**

477 patients were included (baseline phase 287, implementation phase 190). Xpert MTB/RIF had high sensitivity (187/237, 79%, 95% CI: 73–84%) and specificity (190/199, 96%, 95% CI: 92–98%) for culture-positive TB overall, but sensitivity was lower (34/81, 42%, 95% CI: 31–54%) among smear-negative TB cases. Xpert MTB/RIF reduced median days-to-TB detection for all TB cases (1 [IQR 0–26] vs. 0 [IQR 0–1], p<0.001), and for smear-negative TB (35 [IQR 22–55] vs. 22 [IQR 0–33], p = 0.001). However, median days-to-TB treatment was similar for all TB cases (1 [IQR 0–5] vs. 0 [IQR 0–2], p = 0.06) and for smear-negative TB (7 [IQR 3–53] vs. 6 [IQR 1–61], p = 0.78). Two-month mortality was also similar between study phases among 252 TB cases (17% vs. 14%, difference +3%, 95% CI: −21% to +27%, p = 0.80), and among 87 smear-negative TB cases (28% vs. 22%, difference +6%, 95% CI: −34 to +46%, p = 0.77).

**Conclusions:**

Xpert MTB/RIF facilitated more accurate and earlier TB diagnosis, leading to a higher proportion of TB suspects with a confirmed TB diagnosis prior to hospital discharge in a high HIV/low MDR TB prevalence setting. However, our study did not detect a decrease in two-month mortality following implementation of Xpert MTB/RIF possibly because of insufficient powering, differences in empiric TB treatment rates, and disease severity between study phases.

## Introduction

Diagnosis of tuberculosis (TB) is often delayed in resource-limited settings. [1], [2] Factors contributing to the delayed diagnosis of TB include: 1) patient delays, 2) health-system delays and, 3) delays inherent to the conventional TB diagnostic process. [1], [2] Of these, delays related to the low sensitivity of sputum acid-fast bacillus (AFB) smear microscopy, have been identified as the most significant contributor to the total diagnostic delay times experienced by TB patients. [3] The consequences of delayed TB diagnosis and treatment include increased TB-related morbidity, increased mortality, and continued TB transmission. [4], [5] In countries with a high burden of HIV, HIV infection has both reduced the sensitivity of smear microscopy contributing to further delays in TB diagnosis, while simultaneously increasing the urgency in which a rapid TB diagnosis is needed.

Two multi-center studies provide strong evidence that Xpert MTB/RIF (Cepheid, Sunnyvale, CA, USA), the first commercially-available, automated, real-time nucleic-acid amplification test (NAAT) for *Mycobacterium tuberculosis (Mtb)*, could reduce diagnostic delay. [6], [7] Xpert MTB/RIF was highly accurate (including 70% sensitivity in AFB smear-negative patients) and led to more rapid diagnosis and shorter time-to-TB treatment initiation compared to sputum smear microscopy. [6], [7] Xpert MTB/RIF was also simple to perform and required minimal technician training. However, the clinical impact of more rapid TB diagnosis via Xpert MTB/RIF has not been adequately evaluated.

Reducing diagnostic delays may be of particular importance in populations at high risk of early mortality. Although early mortality among hospitalized TB patients in HIV-prevalent settings is high (13–27%) [8]–[13], autopsy studies suggest that TB-attributed mortality may be substantially higher as most patients diagnosed with microbiologically-proven TB postmortem were smear-negative and died prior to TB diagnosis. [12], [14] Thus, we conducted a prospective study to determine whether implementation of Xpert MTB/RIF testing reduces diagnostic delay and improves two-month mortality among hospitalized, predominantly HIV-infected TB suspects.

## Methods

### Overview

A multi-center implementation study of Xpert MTB/RIF performance included summary data on 372 patients from our cohort. [7] Here, we present detailed results on the performance of a single Xpert MTB/RIF test in the full cohort of 477 patients and data on patient outcomes at Mulago Hospital (Kampala, Uganda), the national referral hospital for Uganda.

Study enrollment occurred in two phases, both of which preceded the World Health Organization recommendation endorsing the use of Xpert MTB/RIF. [15] In the baseline phase (August 2009–March 2010), Xpert MTB/RIF results were not reported to clinicians or used for patient management. This phase allowed for the collection of baseline data on study outcomes and was necessary for local validation of Xpert MTB/RIF performance compared with conventional laboratory methods. In the subsequent implementation phase (March 2010–August 2010), Xpert MTB/RIF results were provided to clinicians and were used to inform TB treatment decisions. Pre-defined performance targets approved by the institutional review boards were satisfied before proceeding to the implementation phase. Mulago Hospital ward physicians made all TB treatment decisions during both study phases using all available clinical and laboratory data.

### Ethics Statement

Written informed consent was obtained from all study participants during both study phases. The study was approved by the Makerere University Faculty of Medicine Research Ethics Committee, the Mulago Hospital Institutional Review Board, the Uganda National Council for Science and Technology, and the University of California, San Francisco Committee on Human Research. The study was conducted according to the principles expressed in the Declaration of Helsinki - Ethical Principles for Medical Research Involving Human Subjects.

### Study Population

We enrolled consecutive adults (age ≥18) admitted to Mulago Hospital with cough ≥ two weeks but < six months duration. We excluded patients if they were receiving TB treatment at the time of enrollment, if culture results were unavailable, or if Xpert MTB/RIF was not performed during the implementation phase. In addition, patients who died within three days of hospital admission were excluded from survival analyses because rapid TB diagnosis in this critically-ill group is unlikely to have affected their outcome.

### Patient Evaluation and Follow-up

In both phases, patient evaluation at study enrollment included a standardized demographic and clinical questionnaire, chest radiography, HIV antibody testing and measurement of CD4 cell-count (if HIV-infected), and collection of two spot (day 1) and one early morning (day 2) sputum samples for TB evaluation. A minimum of one mL of sputum per sample was collected. [16] We assessed vital status in all patients either by telephone or in-person at 60 days. We considered patients to be lost to follow-up if they did not return or could not subsequently be contacted within 100 days of their study enrollment. As such, survival analyses are presented to 60 days.

### Sputum Smear Microscopy

Experienced laboratory technicians prepared direct smears from spot and early morning sputum specimens for examination by light-emitting diode-based fluorescence microscopy (LED-FM; Lumin®, LW Scientific, Lawrenceville, GA, USA; magnification 400X) at the Uganda National Tuberculosis Reference Laboratory (NTRL), as previously described. [17] We considered patients to be smear-positive when at least one AFB was seen per high power field (HPF) in at least one sputum smear.

### Xpert MTB/RIF

The GeneXpert platform (Cepheid, Sunnyvale, CA) was installed on an open bench in a separate room in the Mulago Hospital Microbiology Laboratory and Xpert MTB/RIF testing was performed by laboratory technicians who completed a two-day training course. The technicians performed Xpert MTB/RIF directly on a randomly-selected spot sputum specimen as described previously. [18] Of note, all specimens undergoing Xpert MTB/RIF testing were expectorated spontaneously. The technicians printed a formal automated report, which we stored securely during the baseline phase and provided to ward clinicians on the same day during the implementation phase. Xpert MTB/RIF rifampin resistance results were not used for patient management without further confirmation because there were too few rifampin-resistant TB cases to validate diagnostic accuracy in our setting.

### Mycobacterial Culture

NTRL staff cultured one of the two spot specimens and the early morning specimen on Lowenstein-Jensen (LJ) media as previously described. [19] NTRL staff also inoculated sputum sediment from a randomly selected spot specimen into the BACTEC 960 MGIT liquid culture system (Becton Dickinson, Sparks, MD, USA). Cultures were incubated for up to 42 days (MGIT) or 56 days (LJ), and NTRL staff confirmed the identity of any growth by AFB smear microscopy and the Capilia TB speciation assay (TAUNS, Numazu, Japan). We considered patients to have TB if *Mtb* was isolated from any sputum culture.

### Statistical Analysis

We compared categorical variables using the chi-squared or Fisher’s exact test and continuous variables using the Mann–Whitney rank sum test. We calculated point estimates and 95% confidence intervals for the sensitivity, specificity, positive predictive value, and negative predictive value of a single Xpert MTB/RIF test result in reference to mycobacterial culture results (two solid and one liquid culture). In patients with confirmed TB (*i.e.*, at least one positive culture), we calculated the time-to-TB detection as the time from enrollment to first positive result (smear or culture in the baseline phase, and smear, Xpert MTB/RIF, or culture in the implementation phase). We also calculated the time-to-TB treatment as the time from enrollment to treatment initiation. We used Kaplan-Meier survival analysis to estimate the cumulative incidence of survival at two months post-enrollment. We compared the equality of survivor functions for the baseline and implementation phases using the log-rank test, and we estimated 95% confidence intervals using a bootstrapping method with 1000 repetitions for the differences in two-month mortality between study phases. Sample size was determined by enrollment into the multi-center evaluation study [7], and provided 80% power to detect a 10% reduction in two-month mortality in the implementation phase based on a two-sample test of proportions with the level of significance specified in reference to a two-tailed, type I error (p-value) <0.05. We performed all statistical analyses using Stata 11 (Stata Corporation, College Station, TX, USA).

## Results

**Figure 1:**
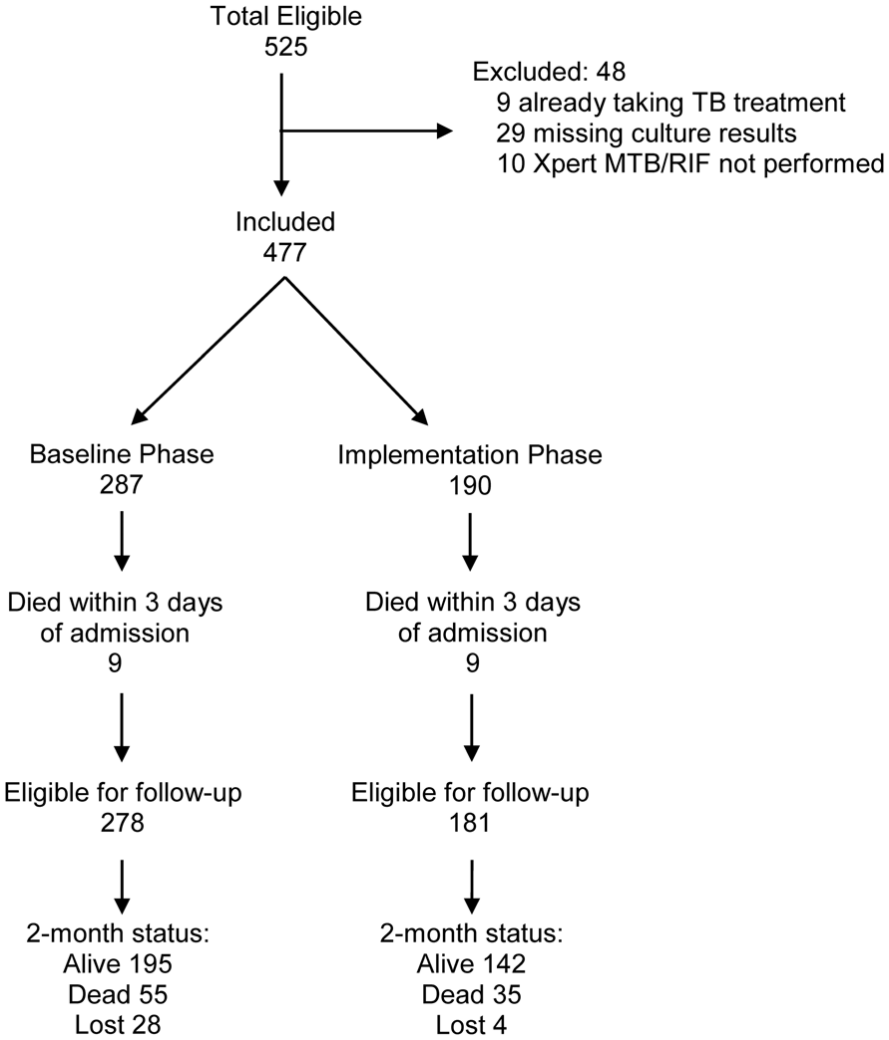
Study enrollment. Of 525 eligible patients, 477 (91%) were included in the study. 287 patients were enrolled during the initial baseline phase (Xpert MTB/RIF results not provided to clinicians during this validation phase) and 190 were enrolled during the implementation phase (Xpert MTB/RIF results provided to clinicians and used for patient management). Eighteen patients (nine in the baseline phase and nine in the implementation phase) died within three days of admission and were not included in survival analysis.

During the study period, 525 eligible adults were enrolled. Nine patients were excluded because they were receiving TB treatment at the time of study enrollment, 29 because culture results were unavailable (25 had insufficient sputum volume for culture, three samples were contaminated, and one sample was lost), and 10 because Xpert MTB/RIF was not performed in the implementation phase (Figure 1). Of the 477 patients included, 287 were enrolled during the baseline phase and 190 during the implementation phase.

### Patient Characteristics

Overall, the study population was 48% female with a median age of 33 years (inter-quartile range [IQR] 27–40; Table 1). Most of the patients in our cohort were infected with HIV (76%) and had advanced HIV-related immunosuppression (median CD4 cell-count 54 cells/µL, IQR 19–157). The prevalence of TB was 40% (189/477) by smear and 55% (262/477) by culture, with no significant differences between the study phases (smear: 37% (107/287) vs. 43% (82/190), p = 0.20; culture: 55% (157/287) vs. 55% (105/190), p = 0.90).

**Table 1:** Demographic and clinical characteristics.

Characteristic, N (%)	Total (N = 477)	Baseline Phase (N = 287)	Implementation Phase (N = 190)	p-value
**Patient characteristics**				
Age (years)*	33 (27–40)	33 (27–40)	31 (27–38)	0.08
Female	229 (48%)	135 (47%)	94 (49%)	0.60
HIV-seropositive	362 (76%)	222 (78%)	140 (74%)	0.32
CD4 count (cells/µL)*	54 (19–157)	51 (19–143)	61 (18–200)	0.41
**Severity of illness**				
Temperature (°C)*	36.8 (36.0–37.9)	36.8 (35.9–38.0)	36.8 (36.3–37.9)	0.19
HR (beats/minute)*	105 (93–120)	104 (91–118)	108 (94–120)	0.24
RR (breaths/minute)*	30 (24–38)	28 (24–36)	32 (28–40)	<0.001
Oxygen saturation (%)*	95 (91–97)	95 (90–97)	95 (92–98)	0.13
Bedbound	12 (3%)	3 (1%)	9 (5%)	0.01
≥1 danger sign present	304 (64%)	168 (59%)	136 (72%)	0.004
**TB characteristics**				
CXR consistent with TB†	293/303 (97%)	171/178 (96%)	122/125 (98%)	0.46
AFB smear-positive	189 (40%)	107 (37%)	82 (43%)	0.20
TB culture-positive	262 (55%)	157 (55%)	105 (55%)	0.90

**Abbreviations:** HR (heart rate); RR (respiratory rate); CXR (chest x-ray); AFB (acid-fast bacilli); TB (tuberculosis).

**Legend:** ^*^Continuous variables presented as medians (interquartile range).

† Admission CXR data available for N = 388.

### Disease Severity

Overall, 64% (304/477) of all patients had danger signs (one or more of the following signs: temperature >39°C, HR >120, RR >30, bedbound), including 57% (52/91) of patients with smear-negative, culture-positive TB. Patients in the implementation phase had evidence of increased disease severity (respiratory rate, bedbound, danger signs) compared to patients in the baseline phase (Table 1).

### Diagnostic Accuracy of Xpert MTB/RIF

A single Xpert MTB/RIF test was performed in 436 patients: 246/287 patients in the baseline phase and all 190 patients in the implementation phase. Xpert MTB/RIF identified 187/237 culture-positive TB cases (sensitivity 79%, 95% CI: 73–84%), including 153/156 smear-positive TB cases (sensitivity 98%, 95% CI: 95–100%) and 34/81 smear-negative TB cases (sensitivity 42%, 95% CI: 31–54%). Sensitivity of Xpert MTB/RIF for TB was similar between the two study phases (baseline phase: 80% [105/132], 95% CI: 72–86%; implementation phase: 78% [82/105], 95% CI: 69–86%; Table 2). Sensitivity of Xpert MTB/RIF for TB was also similar among HIV-infected (79% [144/183], 95% CI: 72–84%) and -uninfected patients (80% [43/54], 95% CI: 67–89%).

**Table 2:** Outcomes in Baseline vs. Implementation phase.

Outcome	Overall	Baseline Phase (N = 246)	Implementation Phase (N = 190)	p-value
Diagnostic Accuracy of Xpert MTB/RIF				
Sensitivity	187/237 (79)	105/132 (80)	82/105 (78)	0.79
	73–84	72–86	69–86	
Specificity	190/199 (96)	107/114 (94)	83/85 (98)	0.20
	92–98	88–98	92–100	
Positive predictive value	95 (92–98)	94 (88–98)	98 (92–100)	–
Negative predictive value	79 (74–84)	80 (72–86)	78 (69–86)	–
Time in Days to…				
TB diagnosis	0 (0–21)	1 (0–26)	0 (0–1)	<0.001
TB treatment	1 (0–4)	1 (0–5)	0 (0–2)	0.06

**Abbreviations:** TB (tuberculosis).

**Legend:** Sensitivity and specificity data are number correct/total (%) 95% CI.

Positive and negative predictive values are presented as % (95% CI).

Times are presented as medians (IQR).

Overall, Xpert MTB/RIF results were negative in 190/199 patients without culture-positive TB (specificity 96%, 95% CI: 92–98%). Specificity was similar in the baseline (94% [107/114], 95% CI: 88–98%) and implementation phases (98% [83/85], 95% CI: 92–100%). Of the nine patients with false-positive Xpert MTB/RIF results, four improved on empiric TB treatment, one improved without TB therapy, and four were lost to follow-up. Specificity of Xpert MTB/RIF was also similar among HIV-infected (95% [137/145], 95% CI: 89–98%) and –uninfected patients (100% [53/53], 95% CI: 93–100%).

### Time-to-TB Detection and Treatment

Among 262 culture-positive TB cases (157 and 105 in the baseline and implementation phases, respectively), the median time-to-detection was shorter in the implementation phase (1 [IQR 0–26; range 0–127] vs. 0 days [IQR 0–1; range 0–55], p = 0.001). Among 189 smear-positive TB patients (107 and 82 in the baseline and implementation phases, respectively), the median time-to-detection was identical, (0 [IQR 0–0; range: 0–3] vs. 0 days [IQR 0–0; range 0–1], p = 0.006), in both study phases. However among 91 smear-negative TB patients (58 and 33 in the baseline and implementation phases, respectively), the median time-to-TB detection was nearly two weeks shorter in the implementation phase (35 [IQR 22–55; range: 12–127] vs. 22 days [IQR 0–33; range: 0–50], p = 0.001). Overall, the proportion of TB patients who were diagnosed by day 1 using smear microscopy in the baseline phase and smear microscopy and/or Xpert MTB/RIF in the implementation phase was significantly increased (55% vs. 78%, p<0.001).

**Figure 2:**
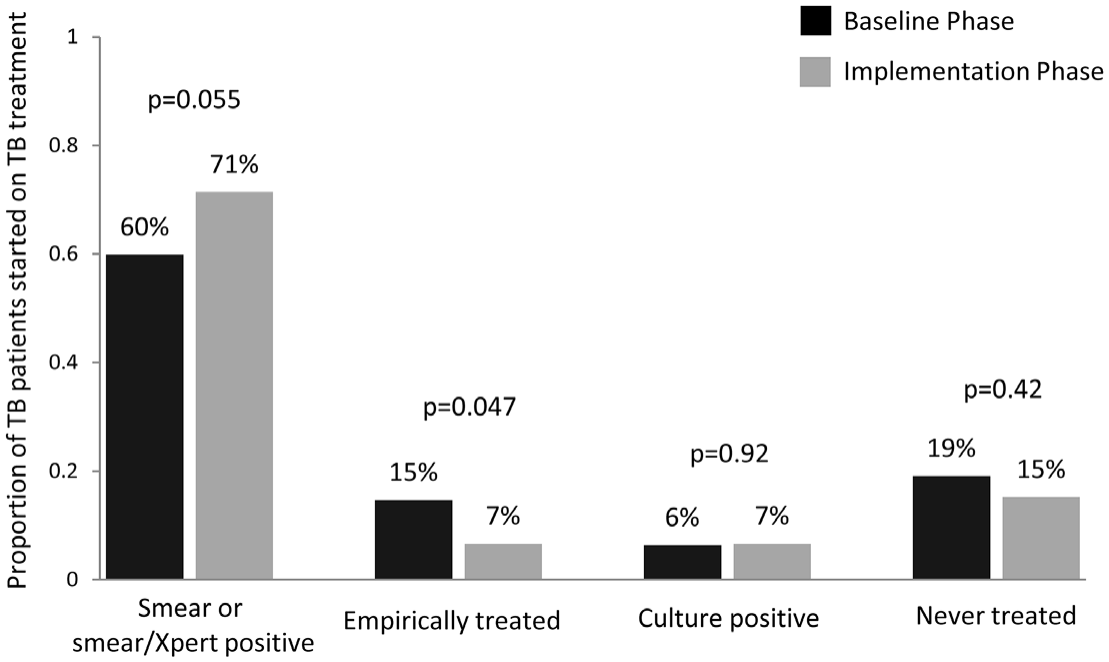
Proportion of TB patients initiated on anti-TB therapy based on test, by study phase. The bars show the proportion of culture-positive TB patients in the baseline vs. the implementation phase started on anti-TB therapy based on rapid test results (*i.e.,* smear in baseline phase and smear/Xpert in implementation phase; 60% vs. 71%, p = 0.055), empirically prior to hospital discharge (15% vs. 7%, p = 0.047), or based on culture results (6% vs. 7%, p = 0.92). The proportion of patients with culture-confirmed TB who were never treated during this study period was similar (19% vs. 15%, p = 0.42).

The median time-to-treatment for all TB patients was longer in the baseline phase relative to the implementation phase (1 [IQR 0–5] vs. 0 days [IQR 0–2], p = 0.06), although this difference was not statistically significant. The median time-to-treatment was also similar between the baseline and implementation phases for smear-negative TB patients (7 [IQR 3–53] vs. 6 days [IQR 1–61], p = 0.78), likely because of different rates of clinician-initiated empiric treatment (*i.e.,* TB treatment initiation despite negative AFB smear results in the baseline phase or negative AFB smear and Xpert MTB/RIF results in the implementation phase). Rates of empiric treatment among patients with culture-confirmed TB were twice as high in the baseline phase as in the implementation phase (15% vs. 7%, p = 0.047; Figure 2). Thus, although the proportion of TB patients initiated on TB treatment by day 1 increased from the baseline phase to the implementation phase (38% vs. 53%, p = 0.02), a similar proportion of patients with culture-positive TB initiated treatment prior to hospital discharge in the two study phases (75% vs. 78%, p = 0.51; Figure 2).

### Two-month Mortality

Overall, there were 64 deaths (22%) in the baseline phase and 44 deaths (23%) in the implementation phase. Of note, in the one-year period prior to the start of the study (August 2008–March 2009), the cumulative incidence of two-month mortality was 21% (95% CI: 16–26) and showed no significant seasonal variation. We excluded 18 patients (nine in the baseline phase and nine in the implementation phase; 3% vs. 5%, p = 0.40) from analyses of two-month mortality because they died within three days of admission. Of note, 10 of the 18 patients excluded from survival analyses had culture-positive TB (four in the baseline phase, six in the implementation phase). Six of the 10 culture-positive TB cases were smear-positive (four in the baseline phase, two in the implementation phase). All four smear-negative culture-positive TB patients were enrolled in the implementation phase: two patients were smear-negative, Xpert-positive while two patients were both smear- and Xpert-negative.

Among the remaining 459 patients, 32 were lost to follow-up (28 in the baseline phase and four in the implementation phase; 10% vs. 2%, p<0.001). There was no difference in median age or median CD4 cell-count between patients who were lost to follow-up versus those patients for whom two-month vital status was known. However patients lost to follow-up were more likely to be male (72% vs. 51%, p = 0.02), less likely to be HIV-infected (56% vs. 76%, p = 0.01), and less likely to have culture-positive TB (38% vs. 57%, p = 0.03).

**Figure 3:**
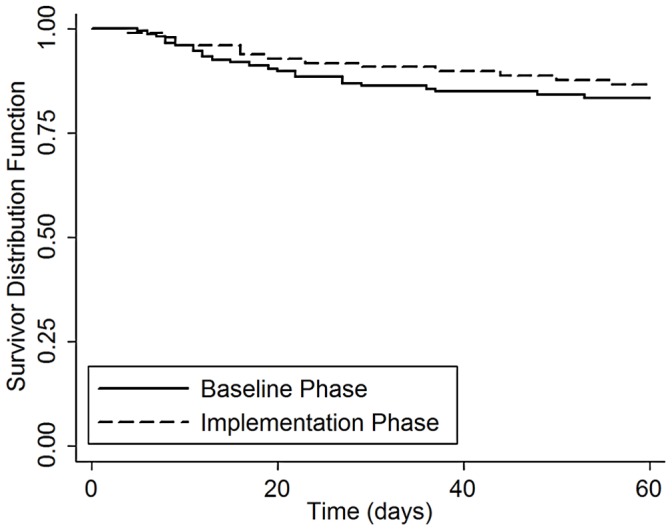
Survival of TB patients with all losses to follow-up censored: Baseline vs. Implementation phase. Kaplan-Meier survival curves are shown for TB patients enrolled during the baseline and implementation phases. There was no difference in two-month mortality by study phase for the 252 patients with culture-positive TB (17% vs. 14%, difference +3%, 95% CI: −21% to +27%, p = 0.80).

**Figure 4:**
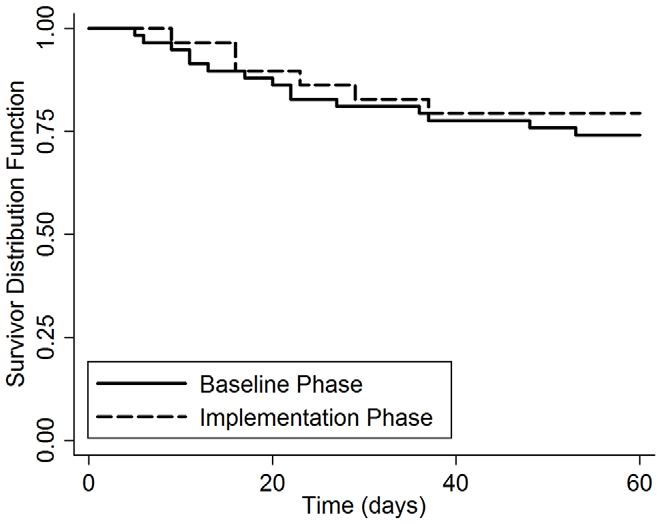
Survival of smear-negative TB patients with all losses to follow-up censored: Baseline vs. Implementation phase. Kaplan-Meier survival curves are shown for smear-negative TB patients enrolled during the baseline and implementation phases. There was no difference in two-month mortality by study phase for the 87 patients with smear-negative, culture-positive TB (28% vs. 22%, difference +6%, 95% CI: −34 to +46%, p = 0.77).

There were 72 deaths among the 427 TB suspects in whom two-month vital status was ascertained, including 42 in the baseline phase and 30 in the implementation phase (Figure 1). The cumulative incidence of two-month mortality was similar in the baseline and implementation phases (17% vs. 17%, difference −0.5%, 95% CI: −18 to +17%, p = 0.96). There was no difference in two-month mortality by study phase for the 252 patients with culture-positive TB (17% vs. 14%, difference +3%, 95% CI: −21% to +27%, p = 0.80; Figure 3) or for the 87 patients with smear-negative, culture-positive TB (28% vs. 22%, difference +6%, 95% CI: −34 to +46%, p = 0.77; Figure 4).

We performed sensitivity analyses to determine the impact of losses to follow-up upon our estimates of two-month mortality. Results were similar when all TB cases lost to follow-up were presumed to be alive (16% vs. 13%, difference +3%, 95% CI: −21% to +26%, p = 0.84; Figure S1) or dead (22% vs. 14%, difference +8%, 95% CI: −15% to +31%, p = 0.53; Figure S2), with no significant difference in two-month mortality by study phase. Similarly, there was no significant difference in two-month mortality by study phase when we included the 18 patients who died within three days of hospital admission (18% vs. 18%, difference −0.6%, 95% CI: −22% to +23%, p = 0.96; Figure S3).

## Discussion

Rapid diagnostics have been predicted to reduce TB-related mortality [20], but few studies have evaluated their impact on patient-important outcomes. [21] In this study, we evaluated metrics of TB diagnosis and two-month mortality before and after implementation of Xpert MTB/RIF in a population at high-risk of poor outcomes. We found that Xpert MTB/RIF was more sensitive than smear microscopy and reduced time-to-TB detection, particularly for smear-negative TB, but did not improve overall time-to-TB treatment or two-month mortality. Although Xpert MTB/RIF is an important advance in TB diagnostics, our results suggest that prompt reporting of smear results and judicious use of empiric treatment may offset its clinical impact in an inpatient setting.

The diagnostic accuracy of Xpert MTB/RIF, the only commercially-available, automated, point-of-care NAAT for *Mtb*, has now been rigorously evaluated in multiple laboratory and clinical settings representing a range of epidemiologic conditions. [22] Sensitivity for smear-negative TB has been variable ranging from 43–83% [22], however, the 42% sensitivity observed in our study is consistent with data from other high HIV-prevalence settings (range 43–61%) employing similar methodologies. [23]–[26] The variable sensitivity for smear-negative TB may reflect differences in definitions of smear-positivity (*i.e.,* classification of smears with >10 AFB per HPF as smear positive), [6], [7] microscopy technique (*i.e.,* light microscopy vs. LED-FM) [6], [7], [27], and/or study populations (*i.e.,* HIV prevalence and severity of illness). [6], [7], [23]–[28] For example, an earlier study including 52 smear-negative TB cases from our cohort reported higher Xpert MTB/RIF sensitivity (58%) when sputum smears with 1–10 AFB per HPF were defined as smear-negative by light microscopy. [7] In contrast, all studies have confirmed that Xpert MTB/RIF has high sensitivity (95–100%) in AFB smear-positive TB cases and high specificity (94–100%) in reference to mycobacterial culture. [6], [7], [23]–[28].

Our study was the first to assess the impact of Xpert MTB/RIF on mortality of TB suspects. While our study was underpowered to detect small differences in two-month mortality between study groups, there are also several potential reasons why two-month mortality may not have decreased following Xpert MTB/RIF implementation. First, reducing delays inherent to the conventional TB diagnostic process through the use of rapid and highly sensitive diagnostics may not improve two-month mortality if these tests are employed after hospitalization. Although our study found that implementation of Xpert MTB/RIF reduces inpatient time-to-TB detection by one day (p<0.001), a systematic review found that TB diagnosis is delayed an average of 68 days after onset of symptoms and 28 days after initial contact with a health-care provider. [1] Thus, improved diagnostic testing at the point of first contact may be needed to prevent TB disease progression and reduce TB-related mortality. Second, Xpert MTB/RIF had lower sensitivity among smear-negative TB patients in our study than in some previous validation studies. [6], [7], [27] We presume that had sensitivity of Xpert MTB/RIF been higher, a higher proportion of patients would have been diagnosed with TB and started on treatment prior to hospital discharge, potentially impacting two-month mortality. Third, a significantly higher proportion of patients in the baseline phase received empiric TB treatment compared to the implementation phase. Early initiation of empiric TB treatment has proven mortality-benefit for seriously-ill, smear-negative TB patients. [29] Lastly, the higher proportion of patients with danger signs in the implementation phase suggests that these patients presented with increased disease severity than those in the baseline phase. Therefore, both higher rates of empiric TB treatment in the baseline phase as well as sicker patients in the implementation phase may have attenuated two-month mortality in this group, potentially masking a significant mortality difference between study phases.

There were several limitations of this study. First, implementation of Xpert MTB/RIF decreased the median time-to-TB detection by only one day (1 vs. 0, p<0.001). Although post-admission delays in TB diagnosis have been associated with early mortality, the median time-to-TB detection in these studies ranged from 4–13 days compared to one day in our study using conventional diagnostics. [13], [30]–[32] Therefore, Xpert MTB/RIF may have a greater impact in settings where smear microscopy results are not provided on the same day. Second, for patients co-infected with HIV, we did not have information of the timing of antiretroviral therapy (ART), which is known to impact mortality in patients with TB. [33]–[35] However, since standard local practice during both study phases was to delay ART for a minimum of four to six weeks after initiation of TB treatment, this is unlikely to have impacted the comparison of two-month mortality between study phases. Third, although we did not provide results of rifampin-resistance testing to ward clinicians, rifampin resistance is uncommon in our setting. Studies from settings with high rates of drug-resistant TB are needed to determine whether earlier identification of MDR TB by Xpert MTB/RIF reduces mortality. Lastly, our study lacked post-mortem data which would have provided more clarity on the causes of death in the study population, and on whether Xpert MTB/RIF reduced TB-specific mortality.

Xpert MTB/RIF is undoubtedly a highly promising candidate for realizing sustained increases in TB case detection and reducing diagnostic delays, including in high HIV-prevalence settings. However the optimal strategy for its implementation in resource-constrained regions remains unclear and is likely to be setting-dependent. Our data suggest that in inpatient settings, where health-system delays in time-to-TB detection are decreased due to the prompt reporting of sputum smear microscopy results and smear-positive patients experience minimal delays in time-to-TB treatment, Xpert MTB/RIF may be reserved for hospitalized smear-negative TB suspects.

While two-month mortality did not improve following implementation of Xpert MTB/RIF in our study, this should not be interpreted as an argument against the widespread adoption of this novel and powerful test. Indeed, upfront Xpert MTB/RIF testing – as a replacement test for smear microscopy – may be particularly important and may significantly improve patient outcomes in settings where: 1) TB suspects first present for care; 2) high-quality smear microscopy results are not provided on the same day; 3) empiric TB treatment is infrequently practiced; and/or 4) drug-resistant TB is prevalent. Furthermore, earlier diagnosis of TB through enhanced diagnostics such as Xpert MTB/RIF identifies patients earlier in their disease course, decreasing both individual morbidity and opportunity for continued TB transmission. [4], [5] However, Xpert MTB/RIF testing alone is unlikely to lead to substantial reductions in TB-related mortality among hospitalized patients in high HIV prevalence settings. Our findings highlight the importance of scaling-up other proven interventions such as early initiation of ART in TB patients co-infected with HIV and of the need for a point-of-care test that enables earlier access to high-quality TB diagnostic testing.

## Supporting Information

Figure S1 **Survival analysis if all losses to follow-up are assumed alive: Baseline vs. Implementation phase.** Kaplan-Meier survival curves are shown for TB patients enrolled during the baseline and implementation phases. There was no difference in two-month mortality by study phase when all TB cases lost to follow-up were presumed to be alive (16% vs. 13%, difference +3%, 95% CI: −21% to +26%, p = 0.84).
(TIF)

Figure S2 **Survival analysis if all losses to follow-up are assumed dead: Baseline vs. Implementation phase.** Kaplan-Meier survival curves are shown for TB patients enrolled during the baseline and implementation phases. There was no difference in two-month mortality by study phase when all TB cases lost to follow-up were presumed to to have died (22% vs. 14%, difference +8%, 95% CI: −15% to +31%, p = 0.53).
(TIF)

Figure S3 **Survival of TB patients (including patients who died within 3 days of admission): Baseline vs. Implementation phase.** Kaplan-Meier survival curves are shown for TB patients enrolled during the baseline and implementation phases. There was no difference in two-month mortality by study phase for the 262 patients with culture-positive TB (18% vs. 18%, difference −0.6%, 95% CI: −22% to +23%, p = 0.96).
(TIF)
