# Supplementary figures and images for: Impact of Xpert MTB/RIF Testing on Tuberculosis Management and Outcomes in Hospitalized Patients in Uganda
Source: PLoS One. 2012 Nov 6;7(11):e48599. doi: 10.1371/journal.pone.0048599 (PMC3490868; doi:10.1371/journal.pone.0048599)

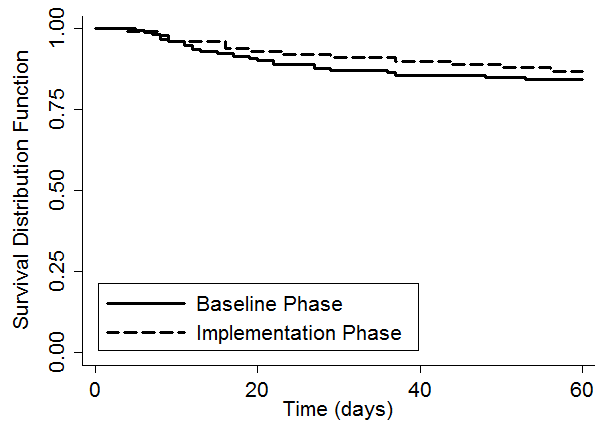

Supplement: Figure S1 — Survival analysis if all losses to follow-up are assumed alive: Baseline vs. Implementation phase. Kaplan-Meier survival curves are shown for TB patients enrolled during the baseline and implementation phases. There was no difference in two-month mortality by study phase when all TB cases lost to follow-up were presumed to be alive (16% vs. 13%, difference +3%, 95% CI: −21% to +26%, p = 0.84). (TIF) [file pone.0048599.s001.tif]

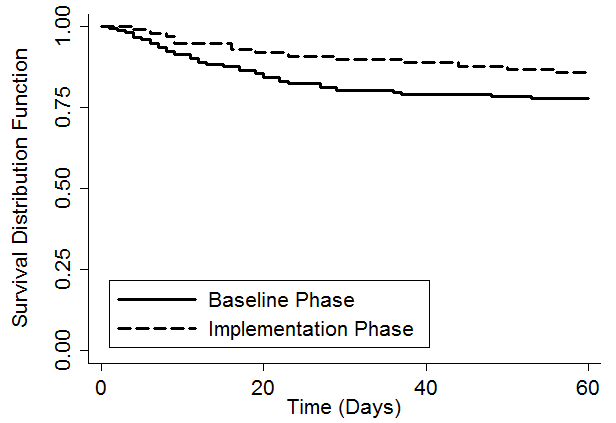

Supplement: Figure S2 — Survival analysis if all losses to follow-up are assumed dead: Baseline vs. Implementation phase. Kaplan-Meier survival curves are shown for TB patients enrolled during the baseline and implementation phases. There was no difference in two-month mortality by study phase when all TB cases lost to follow-up were presumed to to have died (22% vs. 14%, difference +8%, 95% CI: −15% to +31%, p = 0.53). (TIF) [file pone.0048599.s002.tif]

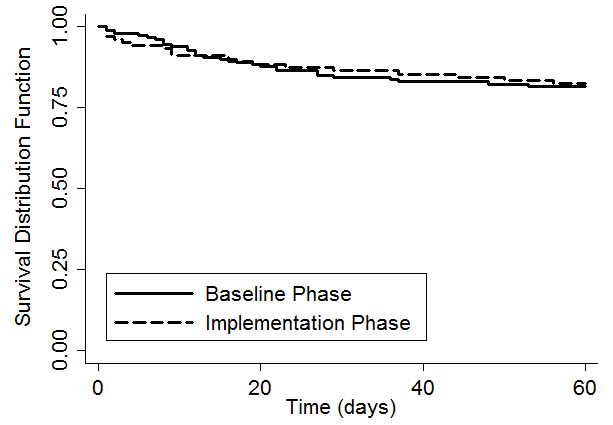

Supplement: Figure S3 — Survival of TB patients (including patients who died within 3 days of admission): Baseline vs. Implementation phase. Kaplan-Meier survival curves are shown for TB patients enrolled during the baseline and implementation phases. There was no difference in two-month mortality by study phase for the 262 patients with culture-positive TB (18% vs. 18%, difference −0.6%, 95% CI: −22% to +23%, p = 0.96). (TIF) [file pone.0048599.s003.tif]
